# Transcatheter closure of perimembranous ventricular septal defects in elderly patients and risk factors of postoperative arrhythmias

**DOI:** 10.3389/fcvm.2025.1580711

**Published:** 2025-07-30

**Authors:** Jianming Wang, Xianyang Zhu, Jingsong Geng, Qiguang Wang

**Affiliations:** Department of Congenital Heart Disease, General Hospital of Northern Theater Command, Shenyang, China

**Keywords:** perimembranous ventricular septal defect, transcatheter closure, septal occluder device, elderly, arrhythmias

## Abstract

**Background:**

The mid- and long-term safety and efficacy of perimembranous ventricular septal defect (pmVSD) closure and the risk factors of postoperative arrhythmias in elderly patients were not known.

**Methods:**

From January 2009 to June 2023, 59 pmVSD elderly patients aged over 60 years were treated through transcatheter intervention. The results and complications of the closure were evaluated by electrocardiography (ECG) and transthoracic echocardiography (TTE) immediately and 1 day after the procedure. TTE was done 1, 3, 6, 12 m, and as a follow up annually.

**Results:**

Interventional closure was successful in all 59 patients. The immediate residual shunt rate was 18.6%. Postoperative arrhythmias occurred in 37 patients (62.7%, 37/59), including severe complications of complete atrioventricular block (cAVB) and implantation of permanent pacemakers in 2 patients (3.4%, 2/59). During the follow-up period, 2 deaths were recorded (due to lung cancer and acute myocardial infarction), and there were no serious complications, such as infective endocarditis, occluder embolism or valve regurgitation requiring surgical treatment. Older age (*P* = 0.006, OR = 1.723, 95% CI: 1.613–1.845) and the occluder size ratio (*d*-value of ventricular septal defect, dVSD/Body Surface Area, BSA) (*P* = 0.002, OR = 1.231, 95% CI: 1.182–1.283) were found to be independent risk factors for a high incidence of arrhythmias after occlusion.

**Conclusions:**

Elderly patients with pmVSD aged over 60 years have a great risk of arrhythmias after transcatheter closure. Older age and the occluder size ratio are associated with short-term postoperative arrhythmias.

## Introduction

Ventricular septal defect (VSD) is the most common congenital heart disease (CHD). The transcatheter closure has been widely performed alternative to surgical repair in strictly selected patients with pmVSDs (1). Due to the variable VSDs and the complexity of the anatomy, transcatheter closure of VSD is more challenging, especially in adults (2–4). Some early untreated pmVSDs still require closure in older adults due to left ventricular (LV) volume overload leading to heart failure, limited activity tolerance, combined pulmonary hypertension or arrhythmia (5, 6). Interventional occlusion of screened adult pmVSD using a domestic modified dual-disk pmVSD occluder and off-label application of the second-generation Amplatzer duct occluder (ADO II) achieved good therapeutic results (7, 8). However, preoperative comorbidities such as valvular disease, arrhythmias, and other cardiovascular pathologies, as well as hypertension and diabetes mellitus, the perioperative challenges and the real benefit of the intervention remains uncertain for transcatheter closure of pmVSDs in the elderly population. Few studies have focused on the indications for interventional occlusion and follow-up outcomes in pmVSD patients older than 60 years. This retrospective study was designed to investigate the safety and efficacy of interventional treatment of patients over 60 years with pmVSDs and to analyse the risk factors of postoperative arrhythmias.

## Materials and methods

### Study design and population

Fifty-nine patients over 60 years of age who underwent transcatheter pmVSD occlusion at the General Hospital of Northern Theater Command between January 2009 and June 2023 were reviewed. The study included 17 male and 42 female congenital pmVSDs patients. According to the consensus of relevant Chinese experts and other literature, the inclusion criteria included (1) the presence of hemodynamic abnormalities, such as left heart volume overload, congestive heart failure, or recurrent endocarditis, etc; (2) the size of the VSD as measured by TTE ≤ 15 mm; (3) the distance from the aortic valve to the upper edge of the defect ≥1 mm; and (4) invasive confirmation of PVR <5WU with pulmonary systemic flow ratio (Qp:Qs) >1.5. The exclusion criteria were as follows: (1) pathological aortic regurgitation; (2) active infective endocarditis; and (3) significant coverage of the tricuspid tendon cords at the edge of the defect. All patients underwent routine coronary angiography before pmVSD occlusion. The Ethics Committee of the General Hospital of Northern Theater Command approved the study (No. XXN-VSD-01), and each patient provided informed written consent before interventional occlusion.

### Pre-procedure planning

Baseline data were collected for all patients. Prior to the procedure, a Holter ECG was evaluated for any rhythm or conduction disturbances. In addition to standard preoperative laboratory assessments, individuals with infections should undergo tests for procalcitonin levels, a respiratory antibody spectrum, blood cultures (both aerobic and anaerobic, in three sets), endotoxin levels, and other relevant infection markers as needed. Patients presenting with heart failure or severe pulmonary arterial hypertension should have NT-ProBNP levels measured and undergo blood gas analysis via the radial or femoral artery. If the etiology of pericardial effusion remains uncertain or the heart failure is not readily explicable, further investigations for tuberculosis, neoplasms, thyroid function, protein levels, and other related parameters should be conducted.

### Occluder devices

**Figure 1:**
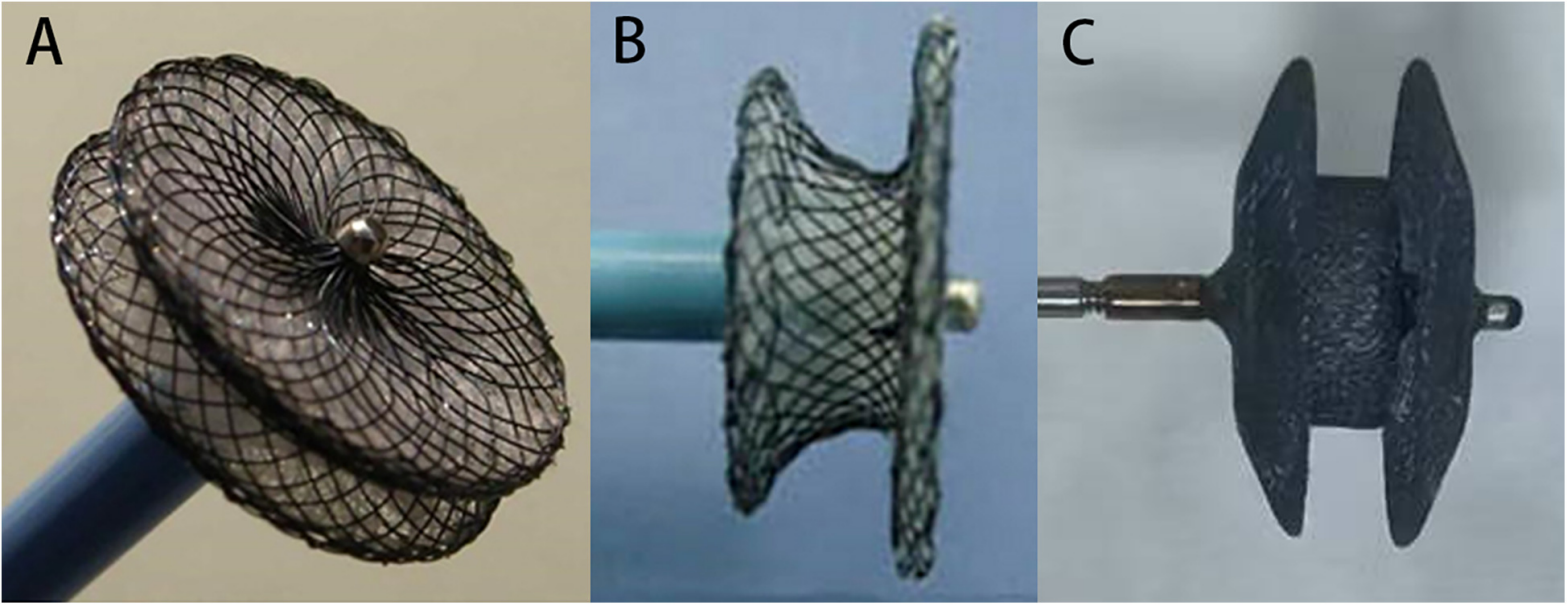
The devices used for pmVSD occlusion. **(A)** The domestic modified symmetrical concentric pmVSD occluder (S-pmVSO). **(B)** The domestic modified asymmetrical concentric pmVSD occluder (AS-pmVSO). **(C)** The second-generation Amplatzer patent ductus arteriosus occluder (ADO II).

In this study, the following domestic modified dual-disk pmVSD occluders were used: a symmetrical concentric pmVSD occluder (S-pmVSO) (Figure 1A) and an asymmetrical concentric pmVSD occluder (AS-pmVSO) (MemoPart Scientech Medical, Shanghai, China) (Figure 1B) (7) The domestically modified double-disk pmVSD occluder is a self-expanding double-disk occluder consisting of a 0.005-inch nickel-titanium alloy wire braid. The diameters of the left and right disks of the S-pmVSO are 4 mm larger than the waist diameter. The diameters of the left and right disks of the AS-pmVSO are 4 and 8 mm larger than the waist diameter, respectively. The waist lengths of the S-pmVSO and the AS-pmVSO are 3 and 2 mm, respectively. The waist lengths of the S-pmVSO are 3 and 2 mm. The occluder lengths range from 4 to 16 mm. The domestic modified double-disk pmVSD occluder and delivery system have been previously described in detail (9, 10). The modified dual-disc pmVSD occluder was approved by the State Food and Drug Administration of China in 2005; it received the European CE mark in 2008.

The ADO II occluder as an “off-label” for pmVSD occlusion used in this study is a second-generation Amplatzer patent ductus arteriosus occluder (Abbott St. Jude Medical, St. Paul, MN, USA) (Figure 1C). The frame is made of a self-expanding, ultrafine nickel-titanium alloy wire braid, polyester fiber patchless design, and the double-disk profile has a smaller concave and more prominent waist structure. The waist diameter has 4 specifications of 3, 4, 5, and 6 mm, and the length of the occluder has 2 specifications of 4 and 6 mm. The delivery sheath is designed with 2 specifications, 4F and 5F, which have better flexibility due to the super elasticity of the occluder, and can be used to pass through complicated and tortuous trails.

When diagnosing a multioutlet membranous aneurysm, the choice of occluder should be determined by the number and size of the VSDs. The AS-pmVSO is capable of closing multiple outlets simultaneously due to its larger left disk. Typically, the suitable size for the occluder exceeds the size of the pmVSD by 1–4 mm. In this study, most patients with aneurysms with multiple-outlet defects were suitable for AS-pmVSO or S-pmVSO occlusion, while patients with small tubular defects, a high risk of cAVB, or complex anatomical defects were suitable for ADO II occluders.

### Procedure

The transcatheter occlusion method for pmVSD has been described in several studies (11). Patients underwent routine coronary angiography before VSD closure. The right heart catheterization examinations were performed during the procedure. Left ventriculography was performed to measure the size of the defect and the distance from the aortic valve. Then the choice of occluder was made for the patients. A S-pmVSD occluder with a distance between the upper edge of the defect and the aortic valve >2 mm was selected, and an AS-pmVSD occluder with a distance between the upper edge of the defect and the aortic valve >4 mm was used in patients with multiple-exit aneurysmal pmVSDs. If the ADO II occluder is used, the waist should be 1–2 mm larger than the narrowest diameter of the defect. The length of the ADO II occluder should match the defect tunnel length. According to the operator's experience and the anatomical characteristics of the ventricular defect, with the principle of minimizing the possibility of complications, the occluder was placed in the center of the defect or at the membranous aneurysm.

### Post-operative care

Heparin, 100 U/kg, was used during the operation, the antibiotic cefuroxime 1.5 bid was applied prophylactically. In our center, if no contraindications were present, a dosage of 40 mg methylprednisolone was administered once daily during the postoperative period and subsequently discontinued after 3–5 days. All postoperative patients were monitored with 48 h rhythm telemetry. Postoperative ECG was performed daily until the seventh day at discharge. All patients underwent TTE and chest radiograph on postoperative day 1.

### Follow-up

Follow-up evaluations included physical examination, ECG, and TTE at postoperative months 1, 3, 6, and 12 and annually thereafter conducted by outpatient physicians. All patients in this study were followed up for at least 6 months. Adverse events were recorded and categorized into major and minor events. Major adverse events included but were not limited to cAVB requiring pacemaker implantation or surgical intervention, death, infective endocarditis, cerebrovascular accident, thromboembolism, or new-onset valve regurgitation requiring surgical repair. These major adverse events were the primary endpoints of this study. Minor adverse events included but were not limited to arrhythmias, new or exacerbated valvular regurgitation < grade 2, mild residual shunts, vascular puncture complications, hemolysis requiring pharmacologic treatment, fever, and rash. An analysis was conducted to identify risk factors associated with postoperative arrhythmia complications. In this context, the term “*d*-value” refers to the difference between the diameter of the occluder's waist and the size of the VSD as measured by left ventriculography. The “occluder size ratio” is defined as the ratio of the *d*-value to the body surface area (BSA).

### Statistical analysis

Continuous variables are expressed as the means ± SDs or medians, and discrete variables are expressed as frequencies or percentages. All the data were analyzed using SPSS 29.0 software (SPSS, Inc., Chicago, Illinois). Count data are expressed as examples (%), and comparisons between groups were made using the chi-square test and Fisher's exact test. An unpaired *t* test was used for comparisons between groups for continuous variables. Variables with statistically significant differences according to the unpaired *t* test were included in a multivariable logistic regression model to analyze the correlation between each factor and arrhythmia after occlusion. Differences were considered statistically significant at *P* < 0.05.

## Results

### Patient characteristics

A total of 59 patients were enrolled in this study, with a mean age of 63.5 ± 3.1 years, and the specific baseline characteristics are summarized in Table 1. Among these patients, 36 had membranous aneurysm-type VSDs. Three patients had a history of infective endocarditis. All patients underwent coronary angiography. Thirty-six patients showed varying degrees of atherosclerotic changes in the coronary arteries, including one 63-year-old woman who showed 85% stenosis of the proximal anterior descending branch of the coronary artery, and the remaining patients had no coronary artery abnormalities. The Holter ECG records showed that there were right bundle-branch block in three patients, frequent premature ventricular beats in three patients, and persistent atrial fibrillation in one patient. Cardiovascular disease comorbidities included hyperlipidemia in 13 patients (22.0%), hypertension in 40 patients (67.8%), and diabetes mellitus in 6 patients (10.2%).

**Table 1:** Baseline characteristics (*n* = 59).

Variable	Values
Sex	
Female	42 (71.2)
Age, years	63.5 ± 3.1
Indications	
Hemodynamic changes (cardiomegaly on chest x-ray, left atrial or left ventricular enlargement verified by echocardiography)	59 (100)
Infectious endocarditis	3 (5.1)
Heart function (New York heart association, NYHA grade)	
II	44 (74.6)
III	15 (25.4)
Echocardiography data	
EF	0.6 ± 0.1
Defect size, mm	5.4 ± 1.4
EDV, ml	98.7 ± 23.2
LAD, mm	43.9 ± 7.1
LVEDD, mm	53.7 ± 4.6
Membranous aneurysm	36 (61.0)
Multioutlet	17 (28.8)
ECG preoperative arrhythmia	7 (11.9)
RBBB	3 (5.1)
Frequent premature ventricular beats	3 (5.1)
Atrial fibrillation	1 (1.7)
Valve regurgitation	18 (30.5)
Mild-moderate mitral valve regurgitation	9 (15.3)
Mild tricuspid valve regurgitation	5 (8.5)
Mild aortic valve regurgitation	4 (6.8)

Values are mean ± SD or *n* (%). VSD, ventricular septal defect; EF, ejection fraction; EDV, end diastolic volume; LAD, left atrial dimension; LVEDD, Left ventricular end-diastolic dimension; ECG, electrocardiography; RBBB, right bundle-branch block.

### Procedure outcomes

**Figure 2:**
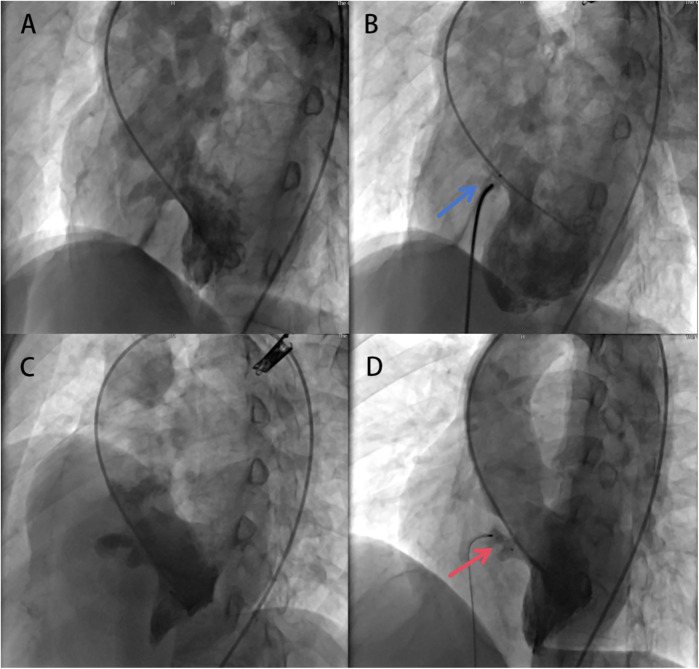
Application of the aS-pmVSO and ADO II occluder for elderly patients with pmVSD occlusion. **(A)** Left ventricular angiography image showing pmVSD with multi-outlet membranous aneurysm, indicating the larger exit position is facing downwards, at a certain distance from the aortic valve. **(B)** After successful implantation of the A4B2 AS-pmVSO, left ventricular angiography image showing trivial residual shunt (blue arrow). **(C)** Left ventricular angiography image showing pmVSD with membranous aneurysm, indicating the exit position is facing upwards, close to the aortic valve. **(D)** After successful implantation of the 9/6ADO II occluder, left ventricular angiography image showing trivial residual shunt (red arrow). pmVSD, perimembranous ventricular defect; S-pmVSO, symmetrical concentric pmVSD occluder.

**Figure 3:**
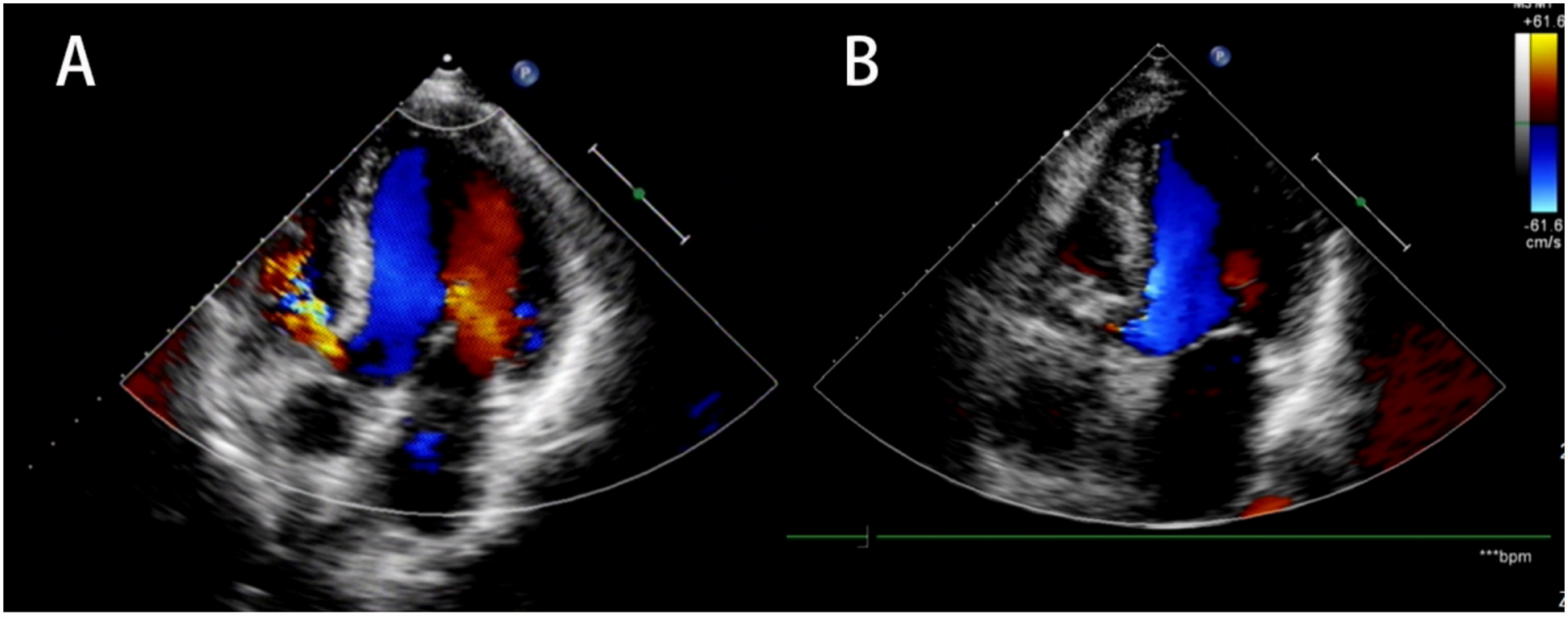
Preoperative and postoperative TTE. **(A)** Preoperative ultrasound evaluation. **(B)** Postoperative TTE reexamination showed optimal placement of the occluder, no residual shunt, and no new onset of aortic or tricuspid regurgitation. TTE, transthoracic echocardiography.

The procedural data are presented in Table 2. All delivery approach were performed by antegrade approach, and established successful arteriovenous circuit. The left to right shunt (Qp/Qs) measured by the right heart catheterization examination was 1.9 ± 0.2. The S-pmVSO (11 patients), AS-pmVSO (32 patients), and the ADO II occluder (16 patients) were used to perform pmVSD closure. The average size of the defect outlet under angiocardiography was 4.8 ± 1.5 mm, the average diameter of the waist of the occluder was 6.9 ± 1.4 mm, the success rate of immediate occlusion was 100%, and the complete occlusion rate was 81.4% (Figure 2). In one patient, cAVB occurred after the initial implantation of an AS-pmVSO, and a normal rhythm was restored after replacement with an ADO II occluder. No patients exhibited new aortic or tricuspid regurgitation on TTE immediately after closure (Figure 3).

**Table 2:** Procedural data (*n* = 59).

Variable	Values
Success rate	59 (100)
Invasive data	
Left-to-right shunt (Qp/Qs)	1.9 ± 0.2
RA mean pressure, mm Hg	6.7 ± 1.7
PA systolic pressure, mm Hg	37.9 ± 8.4
PA diastolic pressure, mm Hg	11.7 ± 5.8
PA mean pressure, mm Hg	22.4 ± 6.7
Pulmonary vascular resistance, woods	2.3 ± 0.8
Size of defect outlet under angiography, mm	4.8 ± 1.5
Device waist diameter, mm	6.9 ± 1.4
Immediate complete closure rate	48 (81.4)
Fluoroscopic time, min	22.5 ± 13.7
Procedure time, min	66.1 ± 26.1
Antegrade approach	59 (100)
Devices used	
S-pmVSO	11 (18.6)
AS-pmVSO	32 (54.2)
ADO II	16 (27.1)

Values are mean ± SD or *n* (%). Qp/Qs, pulmonary systemic flow ratio.

### Postoperative arrhythmia analysis

Postoperative new-onset arrhythmia occurred in 37 patients (62.7%, 37/59). Except for 2 patients with cAVB (3.4%, 2/59), other arrhythmias occurred in 35 patients, including 4 patients with complete left bundle-branch block (6.8%, 4/59); 5 patients with sinus bradycardia (8.5%, 5/59); 2 patients with complete right bundle-branch block with 1 patient with I° atrioventricular block (3.4%, 2/59); 9 patients with frequent premature ventricular beats (15.3%, 9/59) and 8 patients with frequent premature atrial beats (13.6%, 8/59); 6 patients with intermittent junctional rhythm (10.2%, 6/59); and 1 patient with prolonged intraventricular bundle branch conduction (1.7%, 1/59) (Table 3).

**Table 3:** The incidence of arrhythmia and final results during follow-up (*n* = 59).

Postoperative arrhythmia	Number (%)	Final results during follow-up
cAVB	2 (3.4)	In 1 case of cAVB, main 4 mm pmVSD with aneurysm multioutlet, using 6 mm A4B2 AS-pmVSO, implantation of the permanent pacemaker on postoperative 3rd day; the other case of cAVB, 4 mm pmVSD with aneurysm, using 7 mm S-pmVSO, implantation of the permanent pacemaker on postoperative 5th day.
LBBB	4 (6.8)	One case LBBB occurred at postoperative 2nd day, who had sustained LBBB during long term follow-up; one case LBBB occurred at postoperative 3rd day, and returned to normal rhythm at 5th day after occlusion; another one case LBBB occurred at postoperative 4th day, and returned to normal rhythm at 8th day after occlusion.
RBBB	1 (1.7)	Sustained
RBBB with I-AVB	1 (1.7)	Sustained
Sinus bradycardia	5 (8.5)	All occurred immediately, and normal heart rhythm was restored within 3–7 days after occlusion.
Frequent premature ventricular beats	9 (15.3)	Returned to normal or occasional ventricular premature beats at 3–7th day after occlusion.
Frequent premature atrial beats	8 (13.6)	Returned to normal or occasional atrial premature beats at 3–7th day after occlusion.
Accelerated idiojunctional rhythm	6 (10.2)	Normal heart rhythm was restored within 5–7 days after occlusion
Intraventricular conduction delay	1 (1.7)	Occurred at the 2nd day, and returned to normal heart rhythm at 5th day after occlusion

cAVB, complete atrioventricular block; LBBB, left bundle-branch block; RBBB, right bundle-branch block; I-AVB, I° atrioventricular block.

Comparison of general clinical data and occluder parameters between patients in the postoperative arrhythmia group and the postoperative non-arrhythmia group (Table 4): The differences between the postoperative arrhythmia group and the nonpostoperative arrhythmia group were not statistically significant in terms of sex, height, body weight, procedure time, chest-to-cardiac ratio, exposure time, defect size, occluder size, dVSD, VSD diameter/device ratio, or EF (all *P* > 0.05). The arrhythmia events were not related to the type of occluders (*P* = 0.1941), nor *d*-value of VSD among various devices (*P* = 0.730). The ages and dVSD/BSA of patients in the postoperative arrhythmia group were greater than those in the asymptomatic group, and the BSA was smaller than that in the nonpostoperative arrhythmia group (all *P* < 0.05). There was no significant difference in the incidence of other complications between the two groups during the follow-up (Supplementary Table S1).

**Table 4:** Comparison of two groups of patients.

Variable	Postoperative arrhythmia group (*n* = 37)	Postoperative non-arrhythmia group (*n* = 22)	*F* value	*P* value
Age, years	64.8 ± 2.8	60.8 ± 0.8	5.480	0.004
Height, cm	161.8 ± 6.4	165.5 ± 4.8	0.235	0.227
Weight, kg	65.8 ± 2.6	69.2 ± 7.4	3.577	0.159
BSA, m^2^	1.7 ± 0.1	1.8 ± 0.1	0.054	0.037
Procedure time, min	81.8 ± 27.4	57.5 ± 7.6	10.615	0.051
Chest-to-cardiac ratio	0.6 ± 0.1	0.5 ± 0.1	0.265	0.230
Exposure time, min	29.0 ± 15.1	15.7 ± 5.4	8.715	0.055
Defect size, mm	6.0 ± 2.2	5.2 ± 1.6	0.059	0.398
Occulder size, mm	10.1 ± 4.6	9.0 ± 3.2	0.207	0.616
dVSD, mm	4.0 ± 2.8	3.5 ± 2.0	1.339	0.680
dVSD/BSA, mm/m^2^	3.3 ± 1.6	1.9 ± 1.1	1.354	0.032
VSD diameter/device ratio	0.6 ± 0.0	0.6 ± 0.0	0.133	0.407
EF	0.6 ± 0.1	0.6 ± 0.1	0.902	0.241

Values are mean ± SD or *n* (%). BSA, body surface area; dVSD, *d*-value of ventricular septal defect.

Unconditional multivariable logistic regression analysis (Supplementary Table S2) suggested that age (OR = 1.723, 95% CI: 1.613–1.845, *P* = 0.006) and dVSD/BSA (OR = 1.231, 95% CI: 1.182–1.283, *P* = 0.002) were the independent risk factors for arrhythmia in patients who developed arrhythmia after pmVSD closure. However, the BSA was not an independent risk factor (*P* = 0.390).

### Other postoperative follow-up results

All patients were followed up for at least 6 months, with a mean follow-up time of 100.8 ± 30.5 months and a median follow-up time of 76 months (ranging from 6 to 161 months). Of these, 31 patients were followed up for >60 months, and 19 patients were followed up for >120 months.

The incidence of major adverse events was 3.4%. Two patients underwent complete atrioventricular block and implantation of a permanent pacemaker on the 3rd and 5th postoperative days, respectively (3.4%, 2/59). There were no other major adverse events, such as infective endocarditis, embolization of the occluder, or valve regurgitation, requiring surgical treatment for all patients during the follow-up period.

TTE examination revealed that all residual shunts after closure were trivial or small, with an incidence of 18.6% immediately after closure. The residual shunts disappeared on the second postoperative day in 3 patients and at 1 month postoperatively in 7 patients, while a small number of shunts was still present in 1 patient during the follow-up period. Among the 9 patients with mild-moderate mitral valve insufficiency before closure, 3 patients showed mild insufficiency the first day after closure according to TTE examination, and 4 patients showed mild insufficiency 6 months after closure; among the 5 patients with mild tricuspid valve insufficiency and 4 patients with mild aortic valve insufficiency before closure, there was no change in postoperative follow-up. There was no new onset of tricuspid or aortic regurgitation in any of the patients during the follow-up.

Two patients died the 3rd and 6th years after closure (the cause of death was lung cancer and acute myocardial infarction), and the cardiac function of all patients was NYHA grade I or II during the follow-up. After occlusion, no cases of infective endocarditis or cardiovascular or cerebrovascular events were observed during the follow-up period.

## Discussion

Transcatheter pmVSD occlusion for some pediatric or adult patients achieved excellent mid- and long-term follow-up results in several studies (12, 13). In pediatric and young adult pmVSD patients, transcatheter occlusion results were associated with lower mortality and fewer perioperative complications than surgery (14). However, relevant studies and evidence supporting the safety and efficacy of interventional occlusion in elderly pmVSD patients >60 years of age is lacking. Some elderly pmVSD patients may develop several of these complications, such as infective endocarditis, pulmonary hypertension, heart failure due to LV volume overload, and arrhythmia. According to the 2020 ESC Guidelines for the Management of Adult Congenital Heart Disease, in patients with evidence of LV volume overload without pulmonary hypertension, or developed PAH with PVR 3–5 WU, VSD closure is recommended when there is still significant L-R shunt (Qp:Qs > 1.5) (15). Most patients with pmVSD older than 60 years are often comorbid with hypertension, diabetes mellitus, or cerebral infarction, which increases the complexity of treating congenital heart disease. It is controversial whether surgical treatment of these elderly pmVSD patients is associated with a higher risk of trauma and even death, and it is relatively difficult to obtain consensus among elderly patients and physicians (16). However, with the development of interventional therapy, the safety and efficacy of transcatheter treatment for elderly pmVSD patients are of increasing interest.

Aneurysmal pmVSDs are common in elderly pmVSD patients and are often characterized by morphologic diversity (17). The AS-pmVSO has a larger left disk and relatively small right disk, which can close multiple outlets at a time and minimize the risk of tricuspid valve impairment. A thin waist structure can avoid over compression of the defect and the surrounding tissue. The appropriate size of the occluder is usually 1–4 mm larger than the size of the pmVSD from our previous study, and transcatheter closure with a 4-mm waist symmetrical occluder correlated with higher incidences of residual shunts than a 3-mm waist occluder (18). In this study, 17 patients with aneurysms with multiple-outlet defects were suitable for AS-pmVSO or S-pmVSO occlusion, while the other 16 patients with small tubular defects, a high risk of cAVB, or complex anatomical defects were suitable for ADO II occluders. The smaller inner diameter and ultraflexibility of the delivery system with the superelasticity of the occluder make the ADO II more compliant with local anatomic conditions, providing the possibility of more challenging small or complex septal defect occlusions (8, 19). The current rationalization of multiple VSD occluders lays the foundation for successful interventions in elderly pmVSD patients.

When severe pulmonary hypertension occurs, the ventricular septal defect is usually large, and the L-R shunt (Qp/Qs < 1.5) is small. Moreover, it is often accompanied by more severe heart failure. The patients enrolled in this study had relatively small ventricular septal defects and none had severe pulmonary hypertension. However, elderly patients need to be screened for left heart disease-related pulmonary hypertension. There were no pulmonary hypertension contraindications for transcatheter occlusion in the pmVSD patients in this study.

In this study, arrhythmia is the most common complication after interventional occlusion in elderly pmVSD patients, with an incidence of up to 62.7% in this study, including two cases of cAVB, both of which required permanent pacemaker implantation. Most patients who experienced new on-set arrhythmia returned to normal within 1 week after operation, therefore the impact on prognosis may be minimal. In our center, usually methylprednisolone 40 mg was injected intravenously once a day during the postoperative period and was discontinued after 3–5 days. Only 1 patient experienced persistent LBBB, 2 had persistent RBBB, and 2 patients with cAVB required permanent pacemaker implantation. Therefore, patients who did not restore their rhythm in the short term is only 5/59 (8.47%). As aged people, their cardiac function and reserve might be influenced by various cardiovascular diseases, they are vulnerable to interventional treatment, and prone to arrhythmia. Though only cAVB requiring permanent pacemaker implantation as major adverse events, all new postoperative arrhythmias should become a priority concern for elderly pmVSD patients in real world. Multiplex regression analysis revealed that advanced age and the occluder size ratio (difference between occluder and defect size/body surface area) were found to be independent risk factors for a high incidence of arrhythmia after occlusion. Although there was no significant difference between the arrhythmia group and the nonarrhythmia group in terms of operative time, the *P* value was close to 0.05, and there were significantly more operative times in the arrhythmia group than in the nonarrhythmia group in absolute terms, suggesting that shorter operative times may reduce the incidence of postoperative arrhythmias. Electrochemical remodeling of the conduction system due to long-term hemodynamic abnormalities as well as underlying diseases may be the pathophysiological mechanism of arrhythmias after interventional occlusion in elderly pmVSD patients (20, 21). The design and size of the devices were also considered as the main causes of the conduction disturbances (22–24). Considering the small sample size and the fact that most of them were mild postoperative arrhythmias, there was no statistically significant difference for the arrhythmia events using different type of occluders in this study. In the mid- and long-term follow-up, the prognosis of the vast majority of patients improved, but 2 patients died due to underlying disease, which also suggests that we should carefully assess the comprehensive status and prognostic judgment when evaluating the indications for elderly pmVSD patients, adequately balance the risks and benefits, and cautiously apply interventional therapy.

Based on this study, interventional occlusion is effective and feasible for carefully selected elderly pmVSD patients. In 2016, we conducted a study for most younger pmVSD adults revealed that the incidence of arrhythmias was 17.2% (7). In this study, the incidence of arrhythmias after interventional occlusion in elderly pmVSD patients is higher. Advanced age and the occluder size ratio were found to be independent risk factors for a high incidence of arrhythmias after occlusion. In recent research, application of machine learning model can predict postoperative arrhythmias, and aid in risk stratification of patients with pmVSD (25). Interventional occlusion is highly invasive in elderly pmVSD patients, and aggressive treatment is recommended to improve patient prognosis and prevent infective endocarditis. Strict preoperative indications, intraoperative selection of occluder type and close postoperative follow-up are important measures to minimize complications. Randomized controlled studies with larger sample sizes are still needed to further confirm the safety and efficacy of interventional occlusion in elderly pmVSD patients.

### Limitations

This study had several limitations. First, we lacked a controlled cohort study design because it was a retrospective study. Second, this study did not compare surgical therapies nor analyze the occurrence of arrhythmias in the population under 60 years old. Third, patients might not be representative of the general population, as they were from only our center and some of the devices used were not available worldwide. The sample size was relatively small, and a prolonged follow-up period was needed for all patients. Finally, only limited significant differences between groups indicators multivariable logistic regression analysis was conducted, the existence of biases and confounding factors should be considered.

## Conclusion

In conclusion, elderly patients with pmVSD aged over 60 years have a great risk of arrhythmia after transcatheter closure. Older age and the occluder size ratio are associated with short-term postoperative arrhythmias. However, with strict adherence to intervention indications and appropriate occluder selection, severe arrhythmias are rare, and percutaneous closure remains a safe treatment option.

## Data Availability

Data supporting the findings of this study are encrypted and stored in military cardiovascular intervention diagnosis and treatment management information network of China. Requests to access the data should be directed to the corresponding author.
