# Supplementary material for: Transcatheter closure of perimembranous ventricular septal defects in elderly patients and risk factors of postoperative arrhythmias
Source: Front Cardiovasc Med. 2025 Jul 30;12:1580711. doi: 10.3389/fcvm.2025.1580711 (PMC12343494; doi:10.3389/fcvm.2025.1580711)
Supplement: Supplementary file 1 [file Table1.docx]

**Table S1:** Comparison of complications and valve regurgitation prognosis between two groups of patients

| Variable | postoperative Arrhythmia group（n=37） | postoperative Non-arrhythmia group（n=22） | P value |
| --- | --- | --- | --- |
| Residual shunt | 7/37 | 4/22 | >0.999 |
| Prognosis of mitral regurgitation | 2/37 | 0/22 | 0.527 |
| Prognosis of tricuspid regurgitation | 3/37 | 2/22 | >0.999 |
| Prognosis of aortic valve regurgitation | 3/37 | 1/22 | >0.999 |
| Other (vascular complications, rash, and heart failure, etc.) | 3/37 | 1/22 | >0.999 |
