# Supplementary material for: Transcatheter closure of perimembranous ventricular septal defects in elderly patients and risk factors of postoperative arrhythmias
Source: Front Cardiovasc Med. 2025 Jul 30;12:1580711. doi: 10.3389/fcvm.2025.1580711 (PMC12343494; doi:10.3389/fcvm.2025.1580711)
Supplement: Supplementary file 2 [file Table2.docx]

**Table S2:** Results of logistic regression analysis

| Variable | β | Wald | OR | 95% CI | P value |
| --- | --- | --- | --- | --- | --- |
| Age（year，‾X±s） | 0.262 | 29.521 | 1.723 | 1.613, 1.845 | 0.006 |
| BSA (m^2^） | -0.040 | 0.780 | 0.971 | 0.889, 1.060 | 0.390 |
| dVSD/BSA(mm/m^2^） | -0.089 | 12.755 | 1.231 | 1.182,1.283 | 0.002 |
